# *Culex torrentium*: A Potent Vector for the Transmission of West Nile Virus in Central Europe

**DOI:** 10.3390/v11060492

**Published:** 2019-05-29

**Authors:** Stephanie Jansen, Anna Heitmann, Renke Lühken, Mayke Leggewie, Michelle Helms, Marlis Badusche, Giada Rossini, Jonas Schmidt-Chanasit, Egbert Tannich

**Affiliations:** 1 Arbovirology, Bernhard Nocht Institute for Tropical Medicine, 20359 Hamburg, Germany; heitmann@bnitm.de (A.H.); luehken@bnitm.de (R.L.); helms@bnitm.de (M.H.); jonassi@gmx.de (J.S.-C.); 2 Molecular Entomology, Bernhard Nocht Institute for Tropical Medicine, 20359 Hamburg, Germany; leggewie@bnitm.de (M.L.); badusche@bnitm.de (M.B.); 3 German Centre for Infection Research (DZIF), partner site Hamburg-Luebeck-Borstel-Riems, 20359 Hamburg, Germany; tannich@bnitm.de; 4 Unit of Microbiology, Regional Reference Centre for Microbiological Emergencies (CRREM), St. Orsola Malpighi Hospital, 40138 Bologna, Italy; giada.rossini@unibo.it; 5 Faculty of Mathematics, Informatics and Natural Sciences, University of Hamburg, 22609 Hamburg, Germany; 6 Infection Diagnostics Department, Bernhard Nocht Institute for Tropical Medicine, 20359 Hamburg, Germany

**Keywords:** West Nile Virus, *Culex torrentium*, Arbovirus, Vector competence

## Abstract

The continuous circulation of West Nile virus (WNV) in Central, South and East Europe and its recent detection in several dead birds and two horses in Germany highlights the need for information on WNV vector competence of mosquitoes from Central Europe. Therefore, three common *Culex* species (*Culex pipiens* biotype *pipiens*, *Culex pipiens* biotype *molestus* and *Culex torrentium*) from Germany were orally infected with WNV and kept at 18 °C, 21 °C, 24 °C or 27 °C for 14 or 21 days post infection (dpi). Thereafter viable WNV was present in the saliva in all tested taxa, but only at incubation temperatures of 24 °C or 27 °C and predominantly at the extended incubation period of 21 dpi. Highest transmission efficiency rates of 17 % (24 °C) and 24% (27 °C) were found for *Cx. torrentium*. *Culex p. pipiens* and *Cx. p. molestus* showed low transmission efficiencies with a maximum of only 3%. Consequently, temperatures above 21 °C support transmission of WNV, which matches the predominant distribution of human WNV cases around the Mediterranean Sea and in South-East Europe. *Culex torrentium* has been identified as a potent vector for WNV in Central and Northern Europe, which highlights the need for surveillance of mosquito-borne viruses north of the Alps.

## 1. Introduction

West Nile virus (WNV) belongs to the genus *Flavivirus* within the family *Flaviviridae* [1]. WNV is a zoonotic pathogen with an enzootic cycle between mosquitoes as vectors and birds as the primary, amplifying host. Humans, equines and other vertebrates are incidental hosts [2,3]. Human WNV infections can range from asymptomatic or mild clinical symptoms to severe outcomes due to neuroinvasive manifestations [2]. At present, there is no specific treatment or licensed vaccine for human use. WNV has a high epidemic potential as illustrated by its rapid spread after a single introduction to New York City (United States of America) in 1999 [4]. Subsequently, within the following few years, WNV rapidly spread over the North American continent, resulting in more than 20,000 human cases of neuroinvasive diseases and more than 2000 deaths [5]. During recent years, various outbreaks of WNV infections have been reported in central, southern and eastern countries of Europe resulting in several thousand human cases and dozens of fatal outcomes [6].

Mosquito species of the genus *Culex* have a worldwide distribution [7]. They are of medical importance as they can act as vectors for various zoonotic arboviruses from several virus families [8]. The most common *Culex* species in Europe are *Culex torrentium* Martini, 1925 and *Culex pipiens* s.l. L., 1758 [9]. The latter comprises two different biotypes, namely, *Culex pipiens* biotype *pipiens* (*Cx. p. pipiens*) and *Culex pipiens* biotype *molestus* (*Cx. p. molestus*). Species identification of the three taxa is challenging [10,11]. Both biotypes of *Cx. pipiens* s.l. cannot be identified with absolute certainty by morphological criteria and are traditionally classified by biotype-specific mating behaviours, breeding site selection and hibernation habits. *Culex. p. molestus* is considered stenogamous, autogenous, utilizes underground breeding sites and is non-diapausing, while *Cx. p. pipiens* is eurygamous, anautogenous, breeds above the ground and overwinters in diapause. The picture gets even more complex with the occurrence of the sibling species *Cx. torrentium*, because this species resembles *Cx. p. pipiens* morphologically and by its breeding ecology and both species often occur in sympatry [12,13]. *Cx. torrentium* males can be differentiated from *Cx. pipiens* s.l. by characters of the hypopygium, but a reliable morphological differentiation of females is difficult because pre-alar scales easily fall off and the use of morphometric wing characters is generally not established [9,14]. Therefore, species differentiation of the different *Culex* species often relies on molecular identification [10,15]. In Europe, *Cx. pipiens* s.l. and *Cx. torrentium* usually occur together, with *Cx. torrentium* being the dominant species in northern Europe and *Cx. p. pipiens* prevailing in regions south of the Alps [9,16]. In Central Europe such as Austria or Germany, both sister species can be found in sympatry [10,11,12,13].

Due to the wide distribution and high abundance of the three *Culex* taxa in Europe, several studies were performed to analyse their vector capacity for various arboviruses. For many years, it has been assumed, that both *Cx. pipiens* s.l and *Cx. torrentium* are primarily ornithophilic [17], hence they were not classified as important vectors for zoonotic pathogens. However, recent studies demonstrated substantial variability in host feeding patterns of *Cx. p. pipiens* s.l. and *Cx. torrentium*, comprising birds and mammals including humans [10,18,19,20]. Accordingly, the two species have to be considered as bridge vectors for the transmission of zoonotic pathogens from birds to humans. During nationwide monitoring surveys in Germany, different mosquito-borne viruses have been detected in field-collected specimens of the three *Culex* taxa, including Sindbis virus (SINV), Usutu virus (USUV) and Batai virus (BATV) [8]. In addition, there is growing evidence that European populations of both *Cx. pipiens* biotypes and their hybrids are competent vectors for WNV, USUV and SINV [18,21,22,23], but not for Zika virus [24,25] or Chikungunya virus [26]. Concerning *Cx. torrentium*, there is considerably less information about possible vector competences. In a single published study from Germany, it was demonstrated that *Cx. torrentium* is susceptible to WNV infection, but the presence of infectious virus particles in the saliva was not investigated [27]. Other studies from Sweden indicated that *Cx. torrentium* has a higher vector competence for SINV compared to *Cx. pipiens* s.l. [18,28].

The ongoing circulation of WNV in southern and eastern Europe poses a significant risk for the introduction and autochthonous human infections in central and northern Europe, in case suitable vector species and appropriate climatic conditions are present [29,30]. Transmission risk for WNV in Germany was recently demonstrated by the first detection of several WNV-positive birds in summer 2018 [31]. This highlights the need for a temperature-dependent risk assessment of the WNV vector competence for native mosquito vectors in Central Europe. Here we report on experimental WNV infection studies using *Cx. p. pipiens*, *Cx. p. molestus* and *Cx. torrentium* from Germany at different temperature conditions ranging from 18 °C to 27 °C. The results indicate unexpected high WNV transmission rates at 24 °C and 27 °C of 62% and 90%, respectively, but only for *Cx. torrentium*. For a spatial risk assessment, the results were put in relation to temperature data and the actual circulation of WNV in Europe.

## 2. Materials and Methods

### 2.1. Collection and Rearing of Mosquitoes

F0 adults of *Cx. torrentium* and *Cx. p. pipiens* were obtained from egg rafts collected in Langenlehsten, Germany (53°30′ N 10°44′ E) in 2016 and 2017. For *Cx. p. molestus* a labstrain was used, which was established from egg rafts collected in Heidelberg, Germany (49°11′ N 08°39′ E) in 2011. Adult mosquitoes were incubated at 26 °C, with a relative humidity of 80% and a 12:12 light:dark photoperiod. For differentiation of the three *Culex* taxa, DNA was extracted from 1–4 larvae (DNeasy Blood & Tissue Kit, Qiagen, Hilden, Germany) and molecular identification was performed by multiplex quantitative real-time PCR (qRT-PCR) as previously described [11,17].

### 2.2. Experimental Infection and Analysis

Four to 14 days-old female mosquitoes were starved 24 hours before challenged with infectious blood meals containing WNV-1 (clade 1a, strain TOS-09, Genbank HM991273/HM641225, passage 5 from Vero cells) [32] at a final concentration of 10^7^ plaque forming units per milliliter (PFU/mL). This concentration is recommended for artificial blood meals because it corresponds to natural bird viremia [21]. Composition of blood meal was as follows: 50% expired human blood (blood preservation) from human blood bank (not suitable for humans anymore, but useful for mosquitoes), 30% fructose (8% solution), 10% filtrated bovine serum and 10% working solution of the virus stock. Artificial blood meals were provided overnight, using cotton sticks soaked in infectious blood [26]. Subsequently, mosquitoes were anesthetized with CO_2_ and fully engorged females were sorted into a new vial. Mosquitoes were incubated at 80% humidity at 18 °C, 21 °C, 24 °C or 27 °C, respectively. A cotton pad saturated with solution of 8% Fructose was provided during incubation time of 14 or 21 days. 10 randomly selected adult mosquitoes per species were tested by pan-Flavi-, pan-Alpha- and pan-Orthobunyavirus PCRs to test for natural virus infection [33,34,35]. All individuals revealed negative results.

Mosquitoes were analysed for infection rate (IR), transmission rate (TR) and transmission efficiency (TE) 14 and 21 days post infection (dpi) as previously described [25,36]. Infection was investigated by the analysis of extracted RNA (MagMax Pathogen RNA/DNA Kit, Thermo fisher scientific, Waltham, MA, USA) from bodies (excluding legs and wings) according to a previously described qRT-PCR protocol for WNV RNA [27]. The TR was determined by performing a salivation assay for the detection of infectious virus particles as previously described [37]. TR was defined as the number of mosquitoes with WNV-positive saliva per number of WNV-positive mosquito bodies [25,37,38]. TE was calculated as the number of specimens with WNV-positive saliva per total number of fed females [36].

### 2.3. Comparison of the Study Results with Previous Vector Competence Studies and the Actual Circulation of WNV in Europe

The effects of species, temperature and dpi as factors on the IR, TR or TE were tested with generalized linear models with a binomial distribution and logit link function. Where necessary, multi comparison tests between factors were applied depending on the model used with a Tukey matrix of contrast. In addition, TRs in this study were evaluated against the results of previously published studies, conducting WNV infection experiments with European *Culex* spp. mosquito populations. These studies were previously reviewed by Vogels et al. [21] and further updated with a recent study [39]. Furthermore, following the method of Fros et al. [40], temperature data were analyzed to put the vector competence studies in relation to the actual spatial circulation of WNV in Europe. Daily mean temperature data (European re-analysis and observations for monitoring, E-OBS v17.0, available on a 0.25° regular latitude-longitude grid) were downloaded from http://www.ecad.eu [41]. The average daily temperature conditions in July/August for each year between 2011 and 2018 were extracted for all European region on the NUTS 3-level (Nomenclature of Territorial Units for statistic, third level) and visualized in a histogram. In a next step, the number of human WNV cases reported by ECDC for the same statistical regions [6] was linked to the same underlying temperature data. Thereby, the annual averaged July/August temperature data for each reported case were visualized as an additional histogram. Following Fros et al. [40] in order to exclude imported cases, reports of single WNV cases per country and year were eliminated from the analysis. The correlation between the temperature data and human WNV cases was tested with a generalized linear model with a binomial distribution and logit link function. All data analysis and visualizations were conducted with R [42] using the packages cowplot [43], dplyr [44], ggplot2 [45], magrittr [46], maptools [47], raster [48] and multcomp [49].

## 3. Results

All three investigated *Culex* taxa were susceptible to WNV (Table 1), i.e. viral titres of mosquito bodies reached at least the detection limit of the qRT-PCR of 10,000 RNA copies per mosquito specimen. IRs were statistically significant different between the three species (likelihood-ratio test (LR)-χ2 = 0.97662, *df* = 2, *p* < 0.001). *Culex torrentium* had higher IRs compared to *Cx. p. pipiens* and *Cx. p. molestus* (Tukey’s post-hoc tests, *p*  <  0.05), while no differences were found between *Cx. p. pipiens* and *Cx. p. molestus* (Tukey’s post-hoc test, *p*  >  0.05). IRs increased with increasing temperature (LR-χ2 = 0.28262, *df* = 1, *P* < 0.01). Highest IRs of 32% were found for *Cx. torrentium* at an incubation temperature of 27 °C over a period of 14 days dpi, followed by *Cx. p. pipiens* with an IR of 23% (24 °C; 21 dpi). Lowest IRs with a maximum of only 6% were found for *Cx. p. molestus* (27 °C; 21 dpi). In general, IRs were considerably higher at 21 dpi compared to 14 dpi (LR-χ2 = 0.46289, *df* = 1, *P* < 0.001). The only exception was *Cx. torrentium* incubated at 27 °C with a slightly higher IR of 32% at 14 dpi compared to 26% at 21 dpi.

All three *Culex* taxa tested were able to transmit infectious WNV particles, but only at elevated incubation temperatures of 24 °C or 27 °C (LR-χ2 = 4.6129, *df* = 1, *p* < 0.001). In addition, except for *Cx. torrentium* at 27 °C, transmission was only observed for an extended incubation period of 21 dpi compared to 14 dpi (LR-χ2 = 3.9147, *df* = 1, *p* < 0.001, Table 1). TRs differed between the three taxa (LR-χ2 = 2.4958, *df* = 1, *p* < 0.001) with higher values for *Cx. torrentium* compared to the other two species (Tukey’s post-hoc tests, *p*  <  0.001). At 24 °C, TRs for *Cx. p. molestus*, *Cx. p. pipiens* and *Cx. torrentium* were 0%, 14% and 62%, respectively, and at 27 °C TRs were 25%, 33% and 90%, respectively. Accordingly, *Cx. p. molestus* and *Cx. p. pipiens* showed statistically lower TEs of up to a maximum of only 3%, whereas *Cx. torrentium* revealed significant TEs of 17% at 24°C and 24% at 27 °C (LR-χ2 = 0.65335, *df* = 2, *p* < 0.001, Tukey’s post-hoc tests, *p*  <  0.001, Table 1).

**Figure 1:**
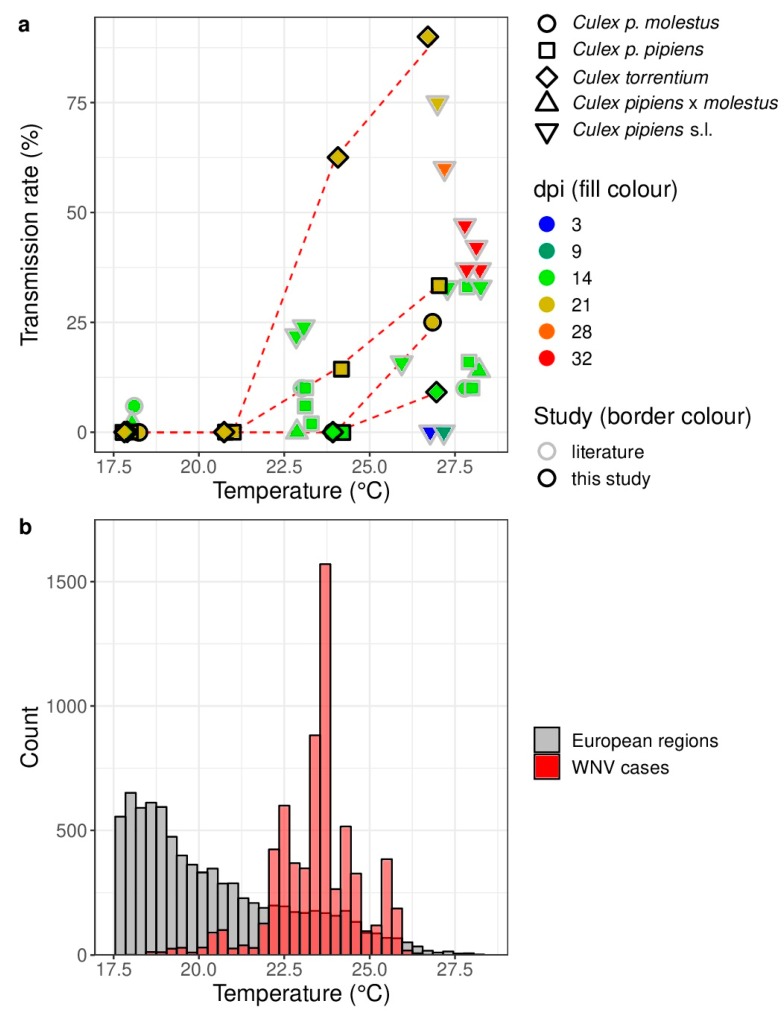
(**a**) Transmission rate of *Culex pipiens* s.l./*torrentium* experimentally infected with West Nile virus at different temperatures and days post infection (dpi) in comparison to previously published vector competence studies with European populations *Culex pipiens* s.l [21]. A small horizontal jitter was added to the points to prevent overlapping.; (**b**) Histograms of the average temperatures in July/August (2011–2018) for all European NUTS 3 (Nomenclature of Territorial Units for Statistics, third level) regions (=gray) and for each detected human WNV case (=red). Count data for the European NUTS 3 regions indicate the cumulative number of times the respective average temperature in July/August was observed over all European NUTS 3 regions for each year from 2011 to 2018. Only the data for the temperatures between 17.5 °C and 28.5 °C are shown.

A distinct temperature dependence of WNV is in line with previous vector competence studies and the distribution of human WNV cases in Europe (Figure 1). Noticeable, transmission rates were only observed for incubation temperatures above 20 °C (Figure 1a). The probability for human WNV cases statistically significant increased with increasing temperature (LR-χ2 = 9769.4, *df* = 1, *p* < 0.001). WNV predominantly circulates in areas with average temperatures above 20 °C during July/August (2011–2018) (Figure 1b).

Similarly, count data for the human WNV cases indicate the cumulative number of times the respective average temperature in July/August was observed for each human WNV case in the respective European NUTS 3 region and year.

## 4. Discussion

Six European mosquito species namely *Aedes albopictus*, *Aedes detritus*, *Aedes japonicus*, *Culex modestus*, *Cx. p. pipiens* and *Cx. p. molestus* are known to be susceptible to WNV infection and able to transmit infectious WNV particles at least under experimental laboratory conditions [21,39]. The study presented here confirms vector competences for *Cx. p. pipiens* and *Cx. p. molestus*, but with moderate transmission rates of 25% and 33%, respectively, and rather low transmission efficiencies up to a maximum of 3%. In addition, we have analyzed the WNV vector competence of *Cx. torrentium*, a further *Culex* species widely distributed and abundant in Central Europe and found unexpected high WNV transmission rates of up to 90% with a calculated maximum transmission efficiency of 24%. Studies specifically addressing WNV transmission of *Cx. torrentium* have not been reported so far. Our findings concerning the two *Cx. pipiens* biotypes are in agreement with previous studies using other European populations of the two biotypes. These studies revealed TRs for WNV of up to 10% for *Cx. p. molestus* and of up to 30% for *Cx. p. pipiens*. Higher TRs of 30%–75% were also reported but in these studies *Cx. pipiens* s.l. mosquitoes were not further differentiated.

Questions remain concerning the mechanism responsible for the high WNV vector competence of *Cx. torrentium* compared to the two *Culex pipiens* biotypes. One thing that is not considered in this study is the number of infectious virus particles that were uptaken by each individual. The number of infectious virus particles per µL will presumably decrease as longer as the blood meal is offered and the time point when each mosquito soaked blood after starting the blood meal was not documented. However, the amount of blood which is ingested by each mosquito varies and therefore the amount of viral particles per fed mosquito varies anyway, which is why this point is not considered in this study. To our knowledge, only one vector competence study has been reported targeting *Cx. torrentium* specifically, indicating that the species is a potent vector of SINV. SINV is a mosquito-borne virus of the genus alphavirus, with a similar ecology as WNV, i.e. enzootic cycle with birds and spill-overs to mammalian species. Experimental infection of birds by either *Cx. torrentium* or *Cx. pipiens* s.l. revealed a noticeable higher vector competence for *Cx. torrentium* [18]. In addition, field studies found highest SINV infection rates for naturally infected *Cx. torrentium* in comparison to *Cx. pipiens* s.l. or *Culiseta morsitans* [28]. Thus, *Cx. torrentium* appears to be a suitable vector for at least two arthropod-borne viruses (arboviruses) from different virus families. Whether the lack of *Wolbachia* as recently reported for *Cx. torrentium* [51] is responsible for this broad vector competence and whether other viruses can be transmitted by *Cx. torrentium* as well, remains to be determined. Host-feeding patterns with nearly equal detections of avian and mammalian hosts [19] indicate that *Cx. torrentium* have to be considered as bridge vector for the transmission of zoonotic pathogens from birds to humans. Thus, the species is considered as the main vector of SINV in Northern Europe and may play a major role in WNV transmission in areas where the species is abundant and respectively favourable environmental conditions are present, in particular, elevated temperatures for an extended time period [9]. The latter may be responsible for the first emergence of WNV in Germany in 2018.

Although intensive surveillance has been conducted during previous years, no WNV circulation was detected in Germany before 2018 [52]. Since the virus is sporadically circulating in neighbouring countries (e.g. France, Czech Republic and Austria) introduction of WNV into Germany has been long-awaited [6]. Nevertheless, intensive screening of birds and mammals in Germany only identified WNV neutralizing antibodies in migratory birds, but none of the animals were positive for WNV RNA [53,54]. Likewise, intensive surveillance of mosquitoes over the last decade confirmed the circulation of various arboviruses in Germany including USUV, SINV and BATV, but did not detect WNV [8]. In contrast, in late summer 2018, several bird specimens from various parts of Germany were tested positive for WNV [31].

This first emergence of WNV in Germany during summer 2018 is clearly linked to temperature anomalies, i.e. significant positive deviation from the long-term mean temperatures, which may have shortened the extrinsic incubation period of WNV [31]. Temperature dependency of WNV replication in the vector has already been discussed [55,56]. Tropical temperatures around 27–28 °C support transmission of WNV, while moderate temperatures of 23–24 °C lead to considerably reduced TRs. In concordance with the results of *Culex* vector competence presented here, other studies also revealed a lack of WNV transmission at lower temperatures (≤21 °C). According to the distribution of human WNV cases in Europe between 2011 and 2018 [6,22] most cases were observed in areas around the Mediterranean Sea and south-eastern countries in Europe, comprising average temperatures between 21 °C and 26 °C in July/August. Thus, although *Cx. torrentium* is a highly competent vector for the transmission of WNV, the main distribution of this species lies within areas of temperate climate in Central and Northern Europe, which in general do not allow transmission of WNV north of the Alps. Temperature conditions in exceptional years, however, may allow WNV circulation even in Central Europe facilitated by the presence of *Cx. torrentium*. This risk may further increase with rising temperatures in the course of climate change. In conclusion, due to the continuing circulation of WNV in Europe and the prevalence of potent vectors for WNV, such as *Cx. torrentium*, a surveillance system that includes birds, mosquitoes and humans should be established or maintained in all European countries to enable early detection and subsequent interventions. This should include areas in Northern and Central Europe, where *Cx. torrentium* is the predominant *Culex* species [9,11]. In addition, due to the high vector competence of *Cx. torrentium* for WNV and SINV [18], further studies should be conducted to evaluate the species’ susceptibility to other arboviruses, such as USUV, which is presently killing thousands of birds in Central Europe [57,58].

## Figures and Tables

**Table 1:** Infection (IR), transmission (TR) and transmission efficiency rates (TE) of three *Culex* species experimentally infected with West Nile virus and kept at four different temperatures, June 2016 to July 2017 (*n* = 788). The experimental analytical sensitivity of the qRT-PCR was analysed according the protocol of Caraguel et al. calculating the limit of detection via endpoint dilution [50]. The limit of detection was defined as 100 copies/µL, corresponding to about 10,000 copies per mosquito specimen. (NA: not analyzed; IR: number of positive saliva/positive bodies; TR: number of positive legs/positive bodies; TE: number of positive saliva per mosquito).

		14 Days Post Infection			21 Days Post Infection		
Mosquito Taxa	T in °C	IR (%)	TR (%)	TE (%)	IR (%)	TR (%)	TE (%)
*Culex p. molestus*	18	0/29 (0.0)	NA	NA	1/29 (3.4)	0/1 (0.0)	NA
	24	0/31 (0.0)	NA	NA	1/31 (3.2)	0/1 (0.0)	NA
	27	0/31 (0.0)	NA	NA	4/62 (6.4)	1/4 (25.0)	1/62 (1.6)
*Culex p. pipiens*	18	1/32 (3.1)	0/1 (0.0)	NA	2/33 (6.1)	0/2 (0.0)	NA
	21	1/30 (3.3)	0/1 (0.0)	NA	3/31 (9.7)	0/3 (0.0)	NA
	24	1/30 (3.3)	0/1 (0.0)	NA	7/31 (22.6)	1/7 (14.3%)	1/31 (3.2)
	27	0/35 (0.0)	NA	NA	3/33 (9.1)	1/3 (33.3)	1/33 (3.0)
*Culex torrentium*	18	2/32 (6.2)	0/2 (0.0)	NA	5/33 (15.2)	0/5 (0.0)	NA
	21	0/31 (0.0)	NA	NA	4/32 (12.5)	0/4 (0.0)	NA
	24	2/31 (6.4)	0/2 (0.0)	NA	8/29 (27.6)	5/8 (62.5)	5/29 (17.2)
	27	11/34 (32.4)	1/11 (9.1)	1/34 (2.9)	10/38 (26.3)	9/10 (90.0)	9/38 (23.7)
